# Genomic contextualisation of ancient DNA molecular data from an Argentinian fifth pandemic *Vibrio cholerae* infection

**DOI:** 10.1099/mgen.0.000580

**Published:** 2021-06-15

**Authors:** Matthew J. Dorman, Nicholas R. Thomson, Josefina Campos

**Affiliations:** ^1^​ Wellcome Sanger Institute, Wellcome Genome Campus, Hinxton, CB10 1SA, UK; ^2^​ Churchill College, Storey’s Way, Cambridge, CB3 0DS, UK; ^3^​ London School of Hygiene and Tropical Medicine, Keppel St., Bloomsbury, London, WC1E 7HT, UK; ^4^​ Instituto Nacional de Enfermedades Infecciosas, INEI-ANLIS “Dr. Carlos G. Malbrán”, Buenos Aires, Argentina

**Keywords:** *Vibrio cholerae*, cholera, fifth pandemic, ancient DNA, aDNA, VCR

## Abstract

Specific lineages of serogroup O1 *Vibrio cholerae* are notorious for causing cholera pandemics, of which there have been seven since the 1800s. Much is known about the sixth pandemic (1899–1923) and the ongoing seventh pandemic (1961–present), but we know very little about the bacteriology of pandemics 1 to 5. Moreover, although we are learning about the contribution of non-O1 non-pandemic *V. cholerae* to cholera dynamics during the current pandemic, we know almost nothing about their role in the past. A recent ancient DNA study has presented what may be the first molecular evidence of a *V. cholerae* infection from the fifth cholera pandemic period (1886–1887 AD) in Argentina. Here, we place the molecular evidence from that study into the genomic context of non-pandemic *V. cholerae* from Latin America and elsewhere, and show that a gene fragment amplified from ancient DNA is most similar to that of *V. cholerae* from the Americas, and from Argentina. Our results corroborate and reinforce the findings of the original study, and collectively suggest that even in the 1880s, non-pandemic *V. cholerae* local to the Americas may have caused sporadic infections in Argentina, just as we know this to have happened during the seventh pandemic in Latin America.

## Data Summary

The authors confirm that all supporting data, code and protocols have been provided within the article or through supplementary data files.

**Fig. 1.**
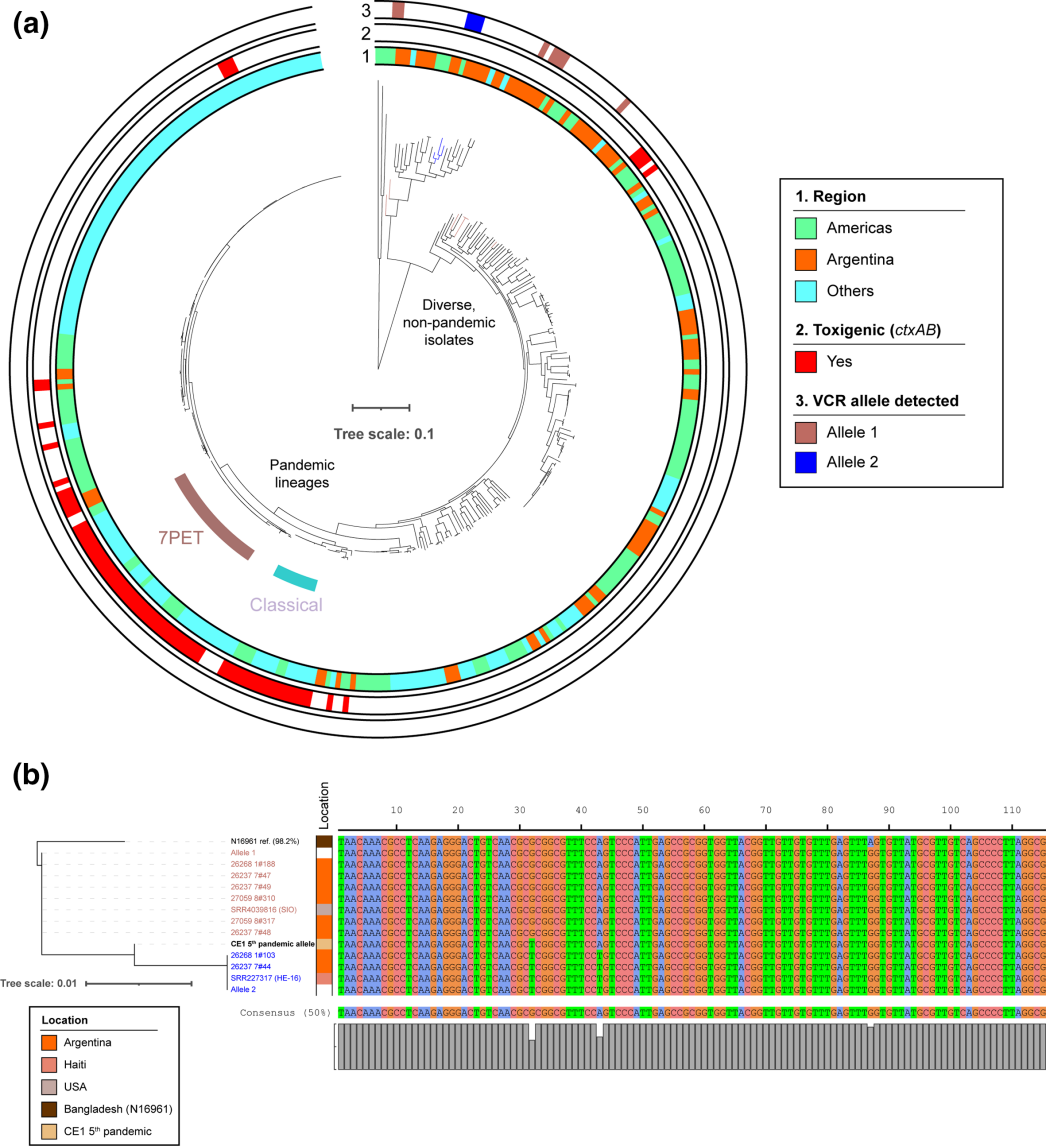
Non-pandemic *V. cholerae* from Argentina and the Americas harbour the most similar VCR alleles to that amplified from aDNA dating from the fifth pandemic. (**a**) A phylogenetic tree [1] containing 380 diverse *V. cholerae* sequences, as well as an outgroup of three *Vibrio* species, on which the tree is rooted. Ten non-O1, non-toxigenic and non-pandemic *V. cholerae* from the Americas harbour VCR alleles most similar to that amplified from the La Zanja CE1 individual (99.13 % nucleotide identity) [2]. All of these are distantly related to the Classical and 7PET pandemic lineages. Bar: Substitutions *per* variable site. (**b**) Two VCR alleles exist amongst the ten isolates highlighted in (**a**) which differ in sequence by 1 nt substitution at position 32 or 43 relative to that of the CE1 amplicon. Sample name colours correspond to the VCR allele identified, as in (**a**). Leaves in (**a**) were coloured manually (Adobe Illustrator CC v23.1.1).

1. No whole-genome sequencing data were generated in this study. Accession numbers for the publicly available sequences collated and/or generated in a previous study [1] and used in this analysis are listed in Table S2 (available in the online version of this article) (https://doi.org/10.6084/m9.figshare.14384225.v1).
2. The collated blastn results used to draw the conclusions in this paper are provided in Table S1 (https://doi.org/10.6084/m9.figshare.14384225.v1).
3. Other metadata, including genome accession numbers and the serogroup for each isolate (simplified to O1 or non-O1/O139), were taken from the supplementary data of Dorman *et al*. [1] and are provided in Table S2 (https://doi.org/10.6084/m9.figshare.14384225.v1).
4. Raw blastn output files, annotated genome assemblies originally collated and used in [1], the VCR query sequence transcribed from Ramirez and colleagues' article [2], and the tree in Fig. 1(b) are provided in a Figshare repository linked to this study: https://dx.doi.org/10.6084/m9.figshare.13636577.
5. The remainder of the data and metadata presented have been published previously under a CC-BY open access licence [1, 3]. The phylogenetic tree presented in Fig. 1(a), and the gene presence/absence matrix used to classify isolates as toxigenic, are available from the Figshare repository linked to Dorman *et al*. [1]: https://doi.org/10.6084/m9.figshare.11310131.

At least seven cholera pandemics have been documented since the 1800s [4–6]. The first six of these are believed to have been caused by serogroup O1 *Vibrio cholerae* of the classical biotype, whereas serogroup O1 biotype El Tor *V. cholerae* is the aetiological agent of the ongoing seventh pandemic (1961–present) [5]. Although much has been learned about the sixth and seventh pandemics from preserved and contemporaneous collections of bacterial cultures, nearly nothing is known about the bacteriology and molecular biology of earlier pandemics. We therefore read with great interest the recent paper by Ramirez and colleagues, in which they present what is believed to be the first genetic and molecular evidence of *V. cholerae* from the fifth cholera pandemic in Argentina (1886–1887 AD) [2].

In their palaeopathological study, Ramirez *et al*. extracted ancient DNA (aDNA) from sediment taken from the pelvic abdominal cavities of four putative cholera victims from the La Zanja archaeological site in Córdoba, Argentina [2]. Procedures were designed to minimise environmental contamination [2]. They managed to amplify a fragment of the *V. cholerae* genome (the *V. cholerae* repetitive DNA sequence, VCR) from two of these aDNA extracts, though they were unable to amplify the *ctxA*, *ctxB* or *tcpA* genes from any of the four specimens studied (these genes are associated with toxigenic, epidemic *V. cholerae*). The authors successfully sequenced the VCR amplicon from one of these samples, and compared this to genome sequences available in GenBank, including partial sequences of two *V. cholerae* isolates from Argentina [2]. The two genomes which contained a VCR sequence most similar to that found in the fifth pandemic Argentinian sample, here dubbed the CE1 allele, were those of Sa5Y and SA3G, non-O1 *V*. *cholerae* isolated in California in 2004 [7].

Together with collaborators, we recently completed a genomic study of the seventh cholera pandemic in Argentina, alongside a simultaneous analysis of non-pandemic *V. cholerae* from the country [1]. For this project, we sequenced 490 Argentinian *V. cholerae*, isolated from 1992 onwards, including 65 non-pandemic isolates. These genome sequences were not included in the analysis of Ramirez *et al*. We speculated that analysing additional genomes from the Americas, and specifically from Argentina, might shed further light on the distribution of this fifth pandemic VCR allele amongst *V. cholerae*. Accordingly, we interrogated the collection of diverse non-pandemic genome assemblies used in our study, a total of 383 genomes (Fig. 1a).

Perhaps unsurprisingly, we could not find a perfect match (100 % nucleotide identity) to the VCR sequence reported by Ramirez *et al*. in any of the genomes in our Argentinian dataset (Table S1). However, ten genomes did contain VCR alleles that differed from the CE1 allele by 1 nt (e-value 2.32×10^−53^; 115/115 aligned nucleotides, 1 nt mismatch, 99.13 % identity, bitscore 203) (Fig. 1a). All of these were isolated in the Americas; eight of the ten are from Argentina, and were isolated in Jujuy, Salta and Formosa provinces, as well as Ciudad Autónoma de Buenos Aires, between 1992 and 2010 [1]. Four of these Argentinian isolates are of clinical origin, and the remainder are environmental isolates [1]. The remaining two genomes are from elsewhere in the Americas: isolate HE-16 from Haiti [8] and isolate SIO from California [9]. Two VCR alleles that differed by 1 nt from the CE1 sequence at one of two different positions were identified (Fig. 1b). The first of these sequences (allele 1, in 7/10 genomes; Fig. 1) was identical to that found in Sa5Y and SA3G by Ramirez *et al*. [2]. The second sequence (allele 2) was found in HE-16 and two Argentinian genomes (Fig. 1). Notably, all ten of these isolates are non-O1 *V. cholerae*, distantly related to pandemic lineages including the Classical lineage, and are non-toxigenic [1] (Fig. 1, Table S2), as the CE1 sample was predicted to be [2].

Although caution must be taken not to over-interpret these data, particularly in the absence of a complete *V. cholerae* genome sequence from this archaeological sample, our genomic observations are consistent with the conclusions made by Ramirez and colleagues – namely, that the individual from whom VCR was amplified and successfully sequenced is likely to have been infected with non-O1 and non-toxigenic *V. cholerae*. This further supports the hypothesis that this Argentinian infection during the fifth pandemic was caused by non-O1 bacteria that are local either to Argentina or to Latin America more generally, rather than being linked to the globally distributed Classical *V. cholerae* lineage that is believed to have caused all historical cholera pandemics prior to the ongoing seventh pandemic [4, 10]. Based on the large number of non-pandemic genomes available to us [1, 11], and because VCR alleles differing from CE1 at two or more positions simultaneously were broadly distributed across *V. cholerae* (Table S1), we speculate that the CE1 VCR allele might be an ancestral form of at least one of the VCR sequences found in contemporary non-pandemic *V. cholerae* local to the Americas. However, a whole genome sequence from this fifth pandemic bacterium would be required to prove whether this sequence is ancestral.

Beyond the curious nature of this archaeological finding, there is a very important subtlety to this observation – although we may still lack molecular or genomic evidence that the Classical lineage caused the fifth cholera pandemic in Argentina and elsewhere, these data suggest that non-pandemic bacteria may have been similarly associated with sporadic infections during the 1880s just as we have found for local lineages of non-pandemic *V. cholerae* in Argentina and Latin America in the present day [1, 11]. While we cannot use this single archaeological sample to draw general conclusions about the fifth cholera pandemic *per se*, it will only be through such investigations that inroads will be made into understanding these historical events. Not only have Ramirez and colleagues presented the first molecular evidence of a *V. cholerae* infection from the fifth pandemic in Latin America, their work is the latest in a recent surge in interest in using the unique aspects of cholera pandemics in Latin America to understand cholera and *V. cholerae* more generally [1, 11, 12]. The precisely defined periods in which pandemic cholera occurred and was introduced to Latin America [4] make this an ideal setting for researching the history of this disease and its epidemiology [1, 11]. This also re-emphasises the importance of aDNA research to studies of historical pandemics [10]. Continued work in this area has the potential to reconstruct the history of previous cholera pandemics, and obtaining partial or whole bacterial genome sequences from aDNA will enable more comprehensive phylogenetic research into these questions.

**Impact Statement**

Cholera is a disease which has been well documented throughout history, due in part to it being highly transmissible and causing explosive epidemics. However, there is a paucity of molecular information about *Vibrio cholerae* pre-dating the turn of the twentieth century. The analysis of ancient DNA (aDNA) is an increasingly common approach by which the histories of bacterial infections can be reconstructed. Ramirez and colleagues recently presented the first aDNA evidence for a *V. cholerae* infection dating from the late 1880s in Argentina – surprisingly, their data suggested infection with a non-toxigenic bacterium. Here, we use a collection of non-pandemic Argentinian genomes to show that the genome fragment amplified by Ramirez *et al*. is most similar to non-pandemic *V. cholerae* from the Americas. Our results strongly indicate that the individual described by Ramirez and colleagues is likely to have been infected with non-pandemic *V. cholerae* local to the Americas. This suggests that non-pandemic *V. cholerae* may have caused sporadic infections in Latin America for hundreds of years. This hints at untapped reserves of information about historical cholera pandemics in Latin America, and emphasises the importance of aDNA research for deriving further insights in this area.

## Methods

### blast analysis and phylogenetics

The 115 nt VCR sequence reported by Ramirez *et al*. [2] was transcribed and used as a query with which to search assembled genome sequences described in Dorman *et al*. [1] using blastn [13] (all of the annotated assemblies used in that study have been uploaded to the Figshare repository supporting this article in GFF3 format). Results were filtered and sorted (cut-offs for inclusion: aligned length ≥100 nt; ordered by bitscore), and are provided in Table S1. The most similar results were defined in line with the results of Ramirez and colleagues [2]: e-value 2.32×10^−53^; 115/115 aligned nucleotides, 1 nt mismatch, 99.13 % identity. The sequences of each result were extracted from the genome assemblies. Two VCR alleles were identified which satisfied these criteria, due to variation at one of two independent nucleotides relative to the reference query. Therefore, both sequences were used along with the best match from the N16961 reference genome [14] to calculate a maximum-likelihood phylogeny under the GTR model using Seaview v4.6.1 and PhyML v3.0 [15, 16], for illustrative purposes. Default settings for maximum-likelihood calculations using nucleotide sequence inputs were used in Seaview v4.6.1. The *V. cholerae* phylogenetic tree presented in Fig. 1(a) has been published previously under a CC-BY 4.0 licence and was re-used *verbatim* in this study [1, 3].

### Data visualisation

Phylogenetic trees were visualised alongside metadata and sequence alignments using the iTOL web service [17]. Isolates were classified as toxigenic on the basis of harbouring both *ctxA* and *ctxB*, as determined from the gene presence/absence matrix in Dorman *et al*. [1, 3]. The figure presented in the paper was edited manually using Adobe Illustrator CC v23.1.1.

## Supplementary Data

Supplementary material 1
